# Mental Wellness and Adherence Self-Efficacy Among Adolescents Living with HIV in the Cape Town Metropole: A Cross-Sectional Survey

**DOI:** 10.3390/pediatric18030073

**Published:** 2026-05-29

**Authors:** Yolanda Mayman, Charné Petinger, Brian van Wyk

**Affiliations:** School of Public Health, University of the Western Cape, Cape Town 7535, South Africa; cpetinger@uwc.ac.za (C.P.); bvanwyk@uwc.ac.za (B.v.W.)

**Keywords:** adolescents, ALHIV, HIV, mental wellness, adherence self-efficacy, ART adherence

## Abstract

Background/Objectives: Adolescents living with HIV (ALHIV) face compounded health and psychosocial challenges while managing lifelong antiretroviral therapy (ART). Mental health difficulties among ALHIV are strongly associated with suboptimal adherence and disengagement from care. While mental illness is well documented, limited empirical evidence exists on the influence of positive mental wellness on adherence self-efficacy among ALHIV. This study assessed mental wellness among ALHIV and identified key psychosocial predictors of adherence self-efficacy in public healthcare facilities in Cape Town, South Africa. Methods: A cross-sectional survey was conducted among ALHIV (*N* = 251) aged 10–19 years who were receiving ART at public healthcare facilities across the Cape Town metropole. Participants completed an electronic questionnaire that assessed ten mental wellness domains and adherence self-efficacy. Descriptive statistics were calculated to summarise participant characteristics and mental wellness scores, while Pearson correlations and multiple linear regression were done to identify associations and independent predictors of adherence self-efficacy using SPSS v29. Results: Most participants were aged 15–19 years (76.9%) and diagnosed with HIV at birth (68.9%). Mental wellness scores were high across all domains (*M* = 3.14–3.71). Hope (*M* = 3.71), spirituality (*M* = 3.58), and purpose in life (*M* = 3.52) were the highest-rated domains. All mental wellness domains were positively correlated with adherence self-efficacy (*p* < 0.001), with the strongest associations being purpose in life (*r* = 0.66), self-acceptance (*r* = 0.66) and resilience (*r* = 0.66). Hope (*p* < 0.001), resilience (*p* = 0.001), purpose in life (*p* = 0.03) and self-acceptance (*p* = 0.012) emerged as significant independent predictors. Conclusions: Positive mental wellness and adolescent-centred psychosocial support in routine HIV care may strengthen adherence self-efficacy and support adolescents’ confidence in managing treatment.

## 1. Introduction

South Africa has the highest prevalence of HIV infection globally, with an estimated 7.8 to 8 million people living with HIV (PLHIV) and more than 5 million receiving antiretroviral therapy (ART) [1,2]. Expanded access to ART has transformed HIV into a manageable chronic condition. However, the long-term success of treatment is dependent on sustained retention in care along with consistent adherence to ART. Within this context, adolescents living with HIV (ALHIV), classified as individuals aged 10 to 19 years, are regarded as a particularly vulnerable group. While adolescents represent the fastest-growing cohort among PLHIV, they continue to experience poorer outcomes across the HIV care continuum when compared to adults. In certain settings, treatment failure among ALHIV has been reported as being up to 50%, which is markedly higher than the 1–15% observed among adult populations living with HIV [3].

Adolescence is widely recognised as a unique formative time and is characterised as a period of multiple physical, emotional and social changes [4,5,6]. There are 1.3 billion adolescents in the world today, making up 16% of the world’s population [4]. Adolescents are generally considered to be a “healthy” segment of the population, and their health-related needs are often overlooked [7]. While all adolescents experience the same difficulties during the period of adolescence, ALHIV experience additional cumulative psychological stressors due to their chronic and often stigmatised condition [7,8,9,10,11]. Globally, it is estimated that one in seven (14.3%) adolescents aged 10–19 years old experience mental health conditions, yet these conditions remain largely undiagnosed and untreated [4]. Studies conducted across sub-Saharan Africa reported a high prevalence of depression, anxiety and post-traumatic stress disorder among ALHIV, with these conditions showing strong associations with suboptimal adherence and viral rebound, as well as an increased risk of mortality [12,13]. Additionally, a recent review reported high prevalence rates of mental health problems among ALHIV, with 30–50% showing symptoms of emotional/behavioural or psychological distress [14].

Mental health has gained global acknowledgement as an integral component of overall health and wellbeing, as evidenced in its inclusion in the Sustainable Development Goals (SDGs) and the World Health Organisation’s (WHO) Mental Health Action Plan (2013–2030) [15,16]. Increasingly, attention has shifted towards mental wellness, which is conceptualised as not merely the absence of mental illness but as a state in which individuals realise their own abilities, are able to cope with everyday stressors, and meaningfully engage with their communities [17,18]. For ALHIV, positive mental wellness functions as a critical protective factor, safeguarding against psychological distress while also supporting the confidence required for lifelong ART adherence [19].

Positive mental wellness is pivotal in adherence self-efficacy in ALHIV. Adherence self-efficacy, defined as the extent or strength of one’s belief in one’s own ability to complete tasks and attain goals despite environmental and social barriers, is increasingly recognised as a key psychosocial mechanism underpinning sustained treatment engagement, optimal adherence and viral suppression [20,21,22,23]. While adherence self-efficacy is related to broader psychosocial functioning, it represents a distinct, treatment-specific construct that represents an individual’s confidence in their ART adherence and is therefore examined as an outcome of mental wellness. It is reported that adolescents who experience higher levels of emotional wellbeing, social connectedness and psychological resilience are more likely to perceive themselves as capable of managing their treatment and coping with stigma [24,25]. Studies from sub-Saharan Africa further highlight the importance of mental wellness domains such as connectedness, hope, future orientation, sense of coherence, self-esteem and self-acceptance in strengthening adherence self-efficacy in ALHIV [26,27,28,29]. Evidence on the importance of mental wellness for adherence self-efficacy remains fragmented, with questions remaining on how multiple domains of mental wellness interact to shape adherence self-efficacy among ALHIV in high-burden settings such as South Africa. This paper reports on mental wellness in ALHIV and identifies key predictors of adherence self-efficacy.

## 2. Materials and Methods

### 2.1. Study Setting

The study was conducted across five purposively selected public healthcare facilities in the Cape Town metropole, spanning primary, secondary, and tertiary levels of care. These facilities were chosen to capture diversity in adolescent HIV service delivery contexts and among ALHIV engaged in care. In 2020, the Cape Town metropole had an estimated 637,353 adolescents aged 10–19 years [30]. By 2022, approximately 540,000 people living with HIV in the metropole were on ART [31].

### 2.2. Study Design

A cross-sectional survey design was used to assess multiple dimensions of mental wellness among ALHIV. Cross-sectional surveys are well-suited for estimating the prevalence of health-related outcomes and identifying associations between variables in a defined population [32]. This design allowed us to examine demographic and clinical factors associated with mental wellness at the time of the study [32,33]. The survey included validated items across ten mental wellness domains and adherence self-efficacy. Data was collected at a single point in time using a structured questionnaire administered electronically during routine clinic visits.

### 2.3. Sampling and Participants

ALHIV aged 10–19 years were recruited from the five participating public healthcare facilities. A facility-based cumulative sampling approach was used, whereby participants were recruited during routine clinic visits. This approach primarily captures adolescents who are engaged in care. Eligible participants were identified by healthcare workers using medical records and clinic club registers. Inclusion criteria were: (1) confirmed HIV-positive status, (2) aged 10–19 years, (3) current enrolment in paediatric, youth, or adult HIV care at one of the selected facilities, and (4) ability to provide informed assent (for participants under 18 years) and/or consent (from parents/guardians or participants aged 18–19 years). Adolescents were approached during routine clinic visits and were enrolled if they voluntarily agreed to participate and provided written informed consent or assent.

According to facility records from a previous study [34] conducted at the same sites, approximately 621 ALHIV were receiving care across the five facilities. A sample size calculation was initially conducted using OpenEpi (Version 3.3) for a finite population. However, given the facility-based cumulative sampling approach, the estimate is interpreted as indicative rather than definitive. The final sample (*n* = 251) was determined by feasibility within the study period and the number of adolescents accessing care at participating facilities during recruitment. Participants were recruited during routine clinic visits and bi-monthly medication collection appointments. Eligible adolescents were identified by healthcare workers using clinic records and ART registers, after which adolescents and caregivers were approached regarding participation. Exact recruitment flow figures (e.g., numbers approached, refused participation, excluded, or unreachable) were not systematically recorded during data collection. For the purposes of regression analysis, the sample size was considered adequate relative to the number of predictors included in the model. With ten independent variables, the sample exceeded recommended minimum ratios of observations per predictor variable and supported the stability of regression estimates.

Recruitment was conducted primarily during bi-monthly medication pick-up visits. Additional efforts were made to include adolescents who had missed scheduled appointments by contacting them telephonically and arranging alternative opportunities to participate.

### 2.4. Data Collection Instrument

The mental wellness instrument used in this study was developed by Orth and van Wyk [19,35] to measure positive mental wellness among adolescents living with HIV (ALHIV) within the South African context. The primary aim of the instrument is to assess multidimensional psychosocial strengths that may support adolescents’ self-management and treatment engagement, rather than focusing solely on symptoms of mental illness. Grounded in the Salutogenic Model of Mental Wellness, the tool evaluates the extent to which adolescents perceive their lives as comprehensible, manageable, and meaningful while living with a chronic condition [11].

The instrument was developed through a multi-phase participatory process to ensure contextual and developmental relevance. This included a systematic review of existing mental health instruments, an integrative review of mental wellness constructs in African settings, and a photovoice study with ALHIV in Cape Town to inform domain selection. Content validity was established through a modified Delphi process with subject matter experts, and face validity was assessed through cognitive interviews with adolescents aged 15–19 years using verbal probing techniques to refine item wording and enhance comprehension [35].

The final instrument comprises 113 items assessing ten mental wellness domains, which include hope, self-acceptance, leisure activities, coping, self-esteem, general self-efficacy, resilience, connectedness, spirituality and purpose in life [35]. In addition, a separate adherence self-efficacy subscale was included as a variable. For the purposes of this study, adherence self-efficacy was analysed as a treatment-specific outcome variable, distinct from the broader mental wellness domains. In this study, the instrument was used to generate composite scores for each domain to examine the relationships between mental wellness dimensions and adherence self-efficacy.

### 2.5. Data Collection Procedures

Data collection was conducted over an eight-month period (from April 2024 to December 2024) by YM and CP. Data were obtained using an electronic questionnaire, which included demographic characteristic items as well as mental wellness items.

The questionnaire was self-administered on a tablet using the RedCap mobile (version 16.0.15) application and was administered in participants’ preferred language (English, Afrikaans, or isiXhosa). The isiXhosa version of the instruments underwent forward translation by a native isiXhosa speaker familiar with the local context and terminology used in adolescent HIV care. The Afrikaans version of the questionnaire was translated in-house by members of the research team who are fluent native Afrikaans speakers. Although the translated versions were reviewed for clarity and contextual appropriateness, formal linguistic validation procedures such as back-translation or certification were not undertaken. During data collection, trained researchers provided additional clarification where necessary to support comprehension and accurate interpretation of questionnaire items, particularly among younger participants (10–14 years). The absence of formal validation procedures for the translated versions may have introduced some measurement variability and is acknowledged as a study limitation.

The mental wellness tool consisted of eleven domains: hope, self-acceptance, leisure activities, coping, self-esteem, self-efficacy, resilience, connectedness, adherence self-efficacy, spirituality and purpose in life. Prior to data collection, this survey tool was piloted amongst participants to ensure content and face validity. Participants answered strongly disagree, disagree, somewhat agree or strongly agree to statements within the various domains. Surveys were administered in private spaces within the healthcare facilities and took approximately 20–30 min to complete. Participants used encrypted electronic tablets and the RedCap software to enter their responses, with data backed up regularly to a secure server to ensure confidentiality and data integrity.

### 2.6. Data Analysis

All data analyses were conducted using IBM SPSS Statistics version 29. A missing values analysis was conducted in SPSS version 29; however, the design of the electronic data collection instrument on REDCap included a condition that the survey could not be completed with any empty fields, therefore eliminating the chance of missing data. Demographic and clinical variables, including age, sex and duration on ART, were considered potential confounders. Due to the exploratory nature of this study, the primary model focused on mental wellness domains. Ten mental wellness domains were analysed as independent variables, with adherence self-efficacy as the dependent variable. Internal consistency of each domain was assessed using Cronbach’s alpha coefficients. Prior to analysis, all items were coded on a four-point Likert scale, ranging from 1 (strongly disagree) to 4 (strongly agree). Composite scores for each mental wellness domain were computed by averaging the items within each domain. Negatively worded items were reverse-coded prior to analysis to ensure consistent directionality of scores. Descriptive statistics were then calculated to summarise adolescents’ levels of mental wellness across the eleven domains. Prior to analysis, assumptions for Pearson correlation and multiple linear regression were assessed. For Pearson correlations, linearity and approximate normality were evaluated using scatterplots and distribution inspection. For multiple linear regression, assumptions of linearity, normality of residuals, homoscedasticity, independence of errors, and the absence of influential outliers were assessed. This was done using residual plots, histograms, normal P-P plots, and standardised residuals. Independence of errors was further evaluated using the Durbin–Watson statistic.

Bivariate analysis was conducted between the domains using Pearson correlation coefficients to explore the interrelationships between domains and to determine which constructs were most strongly associated with adherence self-efficacy. Furthermore, a multiple linear regression analysis was then performed to identify the unique mental wellness predictors of adherence self-efficacy. The composite adherence self-efficacy score served as the dependent variable, and all the remaining mental wellness domains were simultaneously entered as independent variables. Multicollinearity was assessed using variance inflation factors (VIFs) and tolerance statistics. VIF values below 5 and tolerance values above 0.20 were considered acceptable levels of collinearity. Statistical significance for all analyses was set at *p* < 0.05.

## 3. Results

A total of 251 ALHIV participated in the survey. Table 1 illustrates the demographic and treatment characteristics of survey respondents. The majority of participants were aged between 15 and 19 years (76.9%), 58.6% were females, and 68.9% were diagnosed with HIV at birth (Table 1). The mean age of participants was 16.25 years (*SD* = 2.42), indicating that most participants were older adolescents. The majority of participants indicated that they were diagnosed at birth (68.9%) and had been on ART since early childhood (68.9%). Furthermore, most participants reported that they had taken their HIV medication within the past week, reflecting recent medication use (84.9%).

Table 2 below presents the descriptive statistics for the eleven mental wellness domains. Overall, adolescents reported relatively high levels of mental wellness across most constructs, with mean scores ranging from 3.14 to 3.71 on the four-point scale. Hope had the highest mean score (*M* = 3.71, *SD* = 0.43), indicating that adolescents had a general sense of optimism about and hope for their futures, as well as confidence in their ability to achieve their goals. Spirituality (*M* = 3.58; *SD* = 0.54) and purpose in life (*M* = 3.52, *SD* = 0.57) were also among the domains with high mean scores, suggesting that many adolescents draw meaning, motivation and emotional support from their beliefs and long-term aspirations. Adherence self-efficacy similarly showed a high average (*M* = 3.51, *SD* = 0.48), indicating that adolescents reported strong confidence in their ability to adhere to ART.

Domains such as connectedness (*M* = 3.40), leisure activities (*M* = 3.40), coping (*M* = 3.43), and resilience (*M* = 3.38) indicate that while adolescents generally feel supported and able to cope with challenges they may encounter, these areas may still benefit from reinforcement.

Table 3 below presents the Pearson correlations between the eleven mental wellness domains to examine bivariate associations among these constructs and adherence self-efficacy. Adherence self-efficacy refers to an adolescent’s belief in their ability to successfully adhere to treatment plans [19]. All domains were significantly and positively correlated (*p* < 0.001), indicating that higher levels of one mental wellness domain were associated with higher levels in others. Some of the strongest relationships observed were between hope and self-acceptance (*r* = 0.775), coping and leisure activities (*r* = 0.704), resilience and purpose in life (*r* = 0.716), and self-esteem and self-efficacy (*r* = 0.715). These findings suggest that adolescents who are more hopeful and confident and have a sense of purpose tend to demonstrate stronger coping skills, greater resilience, and higher self-regard. Adherence self-efficacy also showed strong correlations with several domains, including purpose in life (*r* = 0.660), self-acceptance (*r* = 0.656), resilience (*r* = 0.656) and hope (*r* = 0.617). This indicates that adolescents who feel confident in managing their ART are more likely to report feeling purposeful, resilient, valued and optimistic.

Table 4, Table 5 and Table 6 below present the results of the multiple linear regression analysis conducted to identify the unique mental wellness predictors of adherence self-efficacy. This allowed for the assessment of the individual contribution of each domain. All ten mental wellness factors were entered simultaneously into the model. The overall model was statistically significant (*p* < 0.001) and explained 57% of the variance in adherence self-efficacy (Adjusted *R*^2^ = 0.576), indicating that the model explains a substantial proportion of variance in adherence self-efficacy.

Four mental wellness domains emerged as significant predictors: hope (*p* = 0.041), self-acceptance (*p* = 0.012), resilience (*p* = 0.003) and purpose in life (*p* = 0.009). These findings suggest that adolescents who report higher levels of hope for the future and feel capable of overcoming challenges are also more confident in managing their ART. Multicollinearity diagnostics showed acceptable levels (VIF range: 2.18–3.27), indicating that collinearity did not substantially bias the regression estimates.

Internal consistency of the mental wellness domains and adherence self-efficacy as an external, dependent variable were assessed using Cronbach’s alpha coefficients. Overall, the domains demonstrated acceptable to excellent reliability, with alpha coefficients ranging from 0.696 to 0.915 (Table 7).

## 4. Discussion

The present study found high levels of adherence self-efficacy as well as generally elevated scores across multiple mental wellness domains, with hope, self-acceptance, and purpose in life scoring the highest. All domains were positively correlated with adherence self-efficacy, with the strongest bivariate associations observed for purpose in life, self-acceptance, resilience and hope. These findings suggest that adherence confidence among adolescents is embedded within a broader network of psychosocial resources rather than being driven solely by treatment knowledge or behavioural competence. This interpretation aligns with evidence that adolescents often conceptualise adherence not merely as a clinical task but as something connected to their aspirations, identity and relational responsibilities [11,36,37].

In the multivariable model, hope and resilience emerged as having the strongest association with adherence self-efficacy, with purpose in life and self-acceptance also remaining statistically significant. Within the Salutogenic Model of Mental Wellness, resilience reflects the dimension of manageability, while hope and purpose represent meaningfulness [19]. Stated more directly, adolescents who believe they have a future worth striving toward and who feel capable of navigating challenges are more confident in their ability to sustain lifelong treatment.

Hope and purpose may be jointly associated with adherence self-efficacy. Hope reflects positive expectations about the future, while purpose in life provides direction and personally valued goals. Together, these constructs may strengthen adherence self-efficacy by linking present-day treatment behaviours to future-oriented outcomes. It is noted, however, that potential interaction effects were not examined in this study. Adolescents who perceive treatment as enabling education, employment, relationships or parenthood may interpret adherence as instrumental to achieving those goals [19,38]. Prior research shows that future orientation and goal-directed thinking are associated with improved engagement in care and persistence with ART among adolescents [27,37]. In this sense, adherence behaviour becomes embedded within a coherent life narrative rather than experienced as an isolated obligation.

Resilience further contributes by strengthening adolescents’ perceived capacity to manage stressors that commonly undermine adherence, including stigma, treatment fatigue, disclosure anxieties and disruptions in routine. In the ALHIV context, resilience has been conceptualised as a dynamic process involving adaptive coping, emotional regulation and the mobilisation of social resources [38,39]. Adolescents who demonstrate higher resilience may be better able to reframe challenges, recover from setbacks and sustain confidence in managing treatment despite psychosocial pressures. Evidence from sub-Saharan Africa indicates that resilience buffers the negative effects of stigma and psychological distress on adherence and viral suppression [12,38]. The current findings support this interpretation, suggesting that resilience strengthens adolescents’ confidence in their capacity to adhere to real-world conditions.

Several domains that were strongly correlated with adherence self-efficacy did not retain significance in the multivariable model, including connectedness, self-esteem and general self-efficacy. This pattern likely reflects conceptual overlap and shared variance across psychosocial constructs. Connectedness and self-esteem may be related to adherence self-efficacy through their associations with other psychosocial constructs such as hope and resilience [19]. However, these relationships were not formally tested in this study. While belonging and peer support are critical for stabilisation and identity development during adolescence, sustained autonomous self-management may depend more strongly on internalised coping capacity and future orientation [37,40]. Thus, the regression findings should not be interpreted as diminishing the importance of social support, but rather as indicating that its effects may be mediated through internal psychosocial strengths. General self-efficacy is broadly related to a sense of personal competence, whereas adherence self-efficacy is related directly to ART adherence-related behaviours.

These findings must also be understood within the broader structural context in which ALHIV live. Across sub-Saharan Africa, stigma remains a significant determinant of mental wellbeing and treatment engagement, often intersecting with poverty, food insecurity and health system constraints [41,42,43]. Limited access to integrated psychosocial services may further weaken adolescents’ capacity to sustain adherence. Evidence suggests that adolescent-friendly services, including peer support groups and structured teen club models, provide protective environments that strengthen belonging, coping capacity and long-term self-management [37,44,45]. The current findings provide quantitative support for these models by identifying the specific psychosocial constructs most closely linked to adherence self-efficacy.

It is important to note that the observed high mean scores across the mental wellness domains suggest the potential for ceiling effects, as responses are clustered toward the upper end of the scale. These high scores may reflect psychosocial strengths but may also be influenced by social desirability bias or contextual factors. Furthermore, the use of a single self-report instrument to predict outcome variables introduces the likelihood of common-method bias, which may further inflate observed associations between the mental wellness domains and adherence self-efficacy. It is for this reason that findings should be interpreted with caution.

### Strengths and Limitations of This Study

This study has several strengths. It is among the first quantitative studies in the South African context to examine multiple dimensions of positive mental wellness simultaneously and assess their independent associations with adherence self-efficacy among adolescents living with HIV. The inclusion of a multidimensional, contextually developed mental wellness instrument allowed for a strengths-based assessment that extends beyond traditional symptom-focused measures. The final sample exceeded the calculated minimum requirement, and the regression model accounted for a substantial proportion of variance in adherence self-efficacy, lending credibility to the stability of the findings within this study population.

Several limitations should also be acknowledged. The cross-sectional design limits the ability to draw causal inferences regarding the directionality of relationships between mental wellness domains and adherence self-efficacy. The reliance on self-reported measures may introduce social desirability bias, particularly in relation to treatment adherence. Participants may have been inclined to report more favourably on their characteristics, as well as report greater confidence in their ability to adhere to treatment. This may have contributed to the high mean scores observed across the domains and an overestimation of the associations between mental wellness and adherence self-efficacy. The sample was clinic-based and predominantly comprised older adolescents, which may limit generalisability to younger adolescents and to those disengaged from care. Additionally, the use of facility-based cumulative sampling limits the generalisability, as the sample represents adolescents in care and not those who are disengaged or experiencing greater psychosocial and adherence challenges. The analyses did not account for clustering by facility, and residual confounding may remain due to unmeasured or unadjusted participant- and site-level factors. Furthermore, although the mental wellness instrument was developed through rigorous participatory and validation processes, the developers acknowledge that ongoing psychometric validation is required to further establish its reliability and construct validity among diverse ALHIV populations [19,35].

## 5. Conclusions

This study contributes to a strengths-based understanding of mental wellness among ALHIV by demonstrating that mental wellness is not simply the absence of mental illness but a multidimensional and relational state that can support self-management and treatment-related confidence. Ultimately, this study demonstrates that positive mental wellness is closely linked to adherence self-efficacy among ALHIV in the Cape Town metropole. Adolescents reported generally high levels of mental wellness, and all domains were positively associated with adherence self-efficacy. These findings suggest that adherence self-efficacy is not only influenced by behavioural skills or treatment literacy but also by adolescents’ capacity to cope with stress, maintain future-oriented goals, and derive meaning in the context of chronic illness. While social connectedness and self-esteem were strongly correlated with adherence self-efficacy, their reduced significance in the multivariable model indicates that these factors may exert their influence indirectly by strengthening more proximal capacities such as resilience and hope. The results underscore the value of integrating strengths-based, adolescent-centred psychosocial support into routine HIV care. Interventions that intentionally foster hope, resilience and purpose, while prioritising self-acceptance, may enhance adolescents’ confidence in sustaining treatment over time and strengthen adherence self-efficacy. Positioning mental wellness as a core component of adolescent HIV care, rather than an ancillary concern, is essential for strengthening adherence self-efficacy and supporting durable, sustained treatment engagement as ALHIV mature into adulthood.

## Figures and Tables

**Table 1:** Demographic and treatment characteristics of adolescents living with HIV on ART in public healthcare facilities in the Cape Town metropole (*N* = 251).

Categories and Sub-Categories	*n*	(%)
Demographic characteristics		
Age (in years)		
10–14	58	23.1
15–19	193	76.9
Sex		
Male	104	41.4
Female	147	58.6
First language		
English	85	33.9
Afrikaans	49	19.5
isiXhosa	117	46.6
Treatment characteristics		
Time of HIV diagnosis		
At birth	173	68.9
After birth	78	31.1
Duration on ART		
Since early childhood	173	68.9
Less than a year	8	3.2
1–5 years	35	13.9
5–10 years	35	13.9
Most recent ART dose (self-reported)		
Less than a week ago	213	84.9
Less than a month ago	24	9.6
More than 3 months ago	7	2.8
More than 6 months ago	7	2.8

Most recent ART dose (self-reported) refers to the most recent time participants reported taking their ART medication. This measure reflects recency of medication intake rather than adherence consistency or missed doses.

**Table 2:** Descriptive statistics of mental wellness domains and adherence self-efficacy.

Domain	Mean	SD	Minimum	Maximum
Hope	3.71	0.43	2.21	4.00
Self-acceptance	3.61	0.51	1.67	4.00
Leisure activities	3.40	0.62	1.30	4.00
Coping	3.43	0.58	1.33	4.00
Self-esteem	3.30	0.55	1.67	4.00
Self-efficacy	3.14	0.58	1.50	4.00
Resilience	3.38	0.53	1.78	4.00
Connectedness	3.40	0.58	1.45	4.00
Spirituality	3.58	0.54	1.00	4.00
Purpose in life	3.52	0.57	1.45	4.00
Adherence self-efficacy	3.51	0.48	1.00	4.00

**Table 3:** Correlations between mental wellness domains (*N* = 251).

Domain	1	2	3	4	5	6	7	8	9	10	11	*p*-Value (With ADH_TOT)
Hope	-	0.775	0.578	0.601	0.453	0.331	0.544	0.383	0.622	0.546	0.617	<0.001
Self-acceptance	0.775	-	0.619	0.650	0.547	0.428	0.605	0.487	0.617	0.620	0.656	<0.001
Leisure activities	0.578	0.619	-	0.704	0.586	0.581	0.626	0.486	0.606	0.652	0.576	<0.001
Coping	0.601	0.650	0.704	-	0.605	0.559	0.677	0.609	0.686	0.639	0.591	<0.001
Self-esteem	0.453	0.547	0.586	0.605	-	0.715	0.638	0.465	0.559	0.681	0.580	<0.001
Self-efficacy	0.331	0.428	0.581	0.559	0.715	-	0.692	0.471	0.496	0.658	0.521	<0.001
Resilience	0.544	0.605	0.626	0.677	0.638	0.692	-	0.650	0.612	0.716	0.656	<0.001
Connectedness	0.383	0.487	0.486	0.609	0.465	0.471	0.650	-	0.592	0.619	0.469	<0.001
Spirituality	0.622	0.617	0.606	0.686	0.559	0.496	0.612	0.592	-	0.648	0.612	<0.001
Purpose in life	0.546	0.620	0.652	0.639	0.681	0.658	0.716	0.619	0.648	-	0.660	<0.001
Adherence self-efficacy	0.617	0.656	0.576	0.591	0.580	0.521	0.656	0.469	0.612	0.660	-	-

**Table 4:** Overall model summary of predictors of adherence self-efficacy among ALHIV.

Model Summary				
Model	R	R-Square	Adjusted R-Square	Std. Error of the Estimate
1	0.770 ^a^	0.593	0.576	0.283

^a^ Predictors (Constant): Purpose in life, hope, connectedness, self-esteem, leisure activities, spirituality, self-efficacy, coping, self-acceptance, resilience.

**Table 5:** ANOVA results for the multiple linear regression model predicting adherence self-efficacy.

Model		Sum of Squares	df	Mean Square	F	Sig.
1	Regression	27.915	10	2.792	34.913	<0.001 ^b^
	Residual	19.190	240	0.080		
	Total	47.105	250			

^b^ Predictors (Constant): Purpose in life, hope, connectedness, self-esteem, leisure activities, spirituality, self-efficacy, coping, self-acceptance, resilience.

**Table 6:** Summary of regression coefficients for the variables predicting adherence self-efficacy.

Model		Unstandardized Coefficients		Standardised Coefficients	t	Sig.	Collinearity Statistics	
		B	Std. Error	Beta			Tolerance	VIF
1	(Constant)	0.283	0.183		1.547	0.123		
	Hope	0.135	0.066	0.146	2.059	0.041	0.336	2.980
	Self-acceptance	0.179	0.070	0.187	2.543	0.012	0.313	3.197
	Leisure activities	0.014	0.063	0.014	0.215	0.830	0.393	2.542
	Coping	−0.009	0.065	−0.010	−0.138	0.891	0.327	3.059
	Self-esteem	0.083	0.068	0.082	1.220	0.224	0.378	2.645
	Self-efficacy	0.017	0.065	0.018	0.265	0.791	0.353	2.836
	Resilience	0.231	0.077	0.222	2.987	0.003	0.306	3.269
	Connectedness	−0.055	0.052	−0.064	−1.056	0.292	0.459	2.178
	Spirituality	0.102	0.053	0.126	1.914	0.057	0.391	2.558
	Purpose in life	0.221	0.084	0.192	2.639	0.009	0.319	3.134

Dependent variable: adherence self-efficacy.

**Table 7:** Psychometric properties of the mental wellness instrument.

Domain	Cronbach’s α
Hope	0.915
Self-acceptance	0.794
Leisure activities	0.746
Coping	0.734
Self-efficacy	0.733
Self-esteem	0.696
Resilience	0.732
Connectedness	0.778
Adherence self-efficacy	0.779
Spirituality	0.808
Purpose in life	0.748

## Data Availability

The datasets analysed in this study are not publicly available due to ethical and privacy restrictions, given the sensitive nature of the data and inclusion of adolescent participants. Further enquiries can be directed to the corresponding author, and data may be made available upon reasonable request, subject to the appropriate ethical approval and data protection requirements.

## Abbreviations

The following abbreviations are used in this manuscript:

ALHIV	Adolescents living with HIV
ART	Antiretroviral therapy
